# Variant c.2158-2A>G in *MANBA* is an important and frequent cause of hereditary hearing loss and beta-mannosidosis among the Czech and Slovak Roma population*-* evidence for a new ethnic-specific variant

**DOI:** 10.1186/s13023-020-01508-3

**Published:** 2020-08-26

**Authors:** Dana Safka Brozkova, Lukas Varga, Anna Uhrova Meszarosova, Zuzana Slobodova, Martina Skopkova, Andrea Soltysova, Andrej Ficek, Jan Jencik, Jana Lastuvkova, Daniela Gasperikova, Pavel Seeman

**Affiliations:** 1 DNA laboratory, Department of Paediatric Neurology, Second Faculty of Medicine, Charles University and Motol University Hospital, Prague, Czech Republic; 2 Department of Otorhinolaryngology – Head and Neck Surgery, Faculty of Medicine and University Hospital, Comenius University, Bratislava, Slovakia; 3 Diabgene Laboratory, Institute of Experimental Endocrinology, Biomedical Research Center, Slovak Academy of Sciences, Bratislava, Slovakia; 4 Department of Molecular Biology, Faculty of Natural Sciences, Comenius University, Bratislava, Slovakia; 5 Institute for Clinical and Translational Research, Biomedical Research Center, Slovak Academy of Sciences, Bratislava, Slovakia; 6 Department of Medical Genetics, Masaryk Hospital in Usti nad Labem, Regional Health Corporation, Usti nad Labem, Czech Republic

**Keywords:** Beta-mannosidosis, Ethnic-specific variant, Hearing loss, Roma, Mental retardation

## Abstract

**Background:**

The Roma are a European ethnic minority threatened by several recessive diseases.

Variants in *MANBA* cause a rare lysosomal storage disorder named beta-mannosidosis whose clinical manifestation includes deafness and mental retardation. Since 1986, only 23 patients with beta-mannosidosis and biallelic *MANBA* variants have been described worldwide.

**Results:**

We now report on further 10 beta-mannosidosis patients of Roma origin from eight families in the Czech and Slovak Republics with hearing loss, mental retardation and homozygous pathogenic variants in *MANBA*.

*MANBA* variant c.2158-2A>G screening among 345 anonymized normal hearing controls from Roma populations revealed a carrier/heterozygote frequency of 3.77%. This is about 925 times higher than the frequency of this variant in the gnomAD public database and classifies the c.2158-2A>G variant as a prevalent, ethnic-specific variant causing hearing loss and mental retardation in a homozygous state. The frequency of heterozygotes/carriers is similar to another pathogenic variant c.71G>A (p.W24*) in *GJB2*, regarded as the most frequent variant causing deafness in Roma populations.

**Conlcusion:**

Beta-mannosidosis, due to a homozygous c.2158-2A>G *MANBA* variant, is an important and previously unknown cause of hearing loss and mental retardation among Central European Roma.

## Background

Beta-mannosidosis is an extremely rare metabolic storage disorder resulting from a beta-mannosidase deficiency that is involved in the degradation of glycoproteins. (MANSB [MIM: 248510]). The rarity of the disease has been documented in only 23 reported affected patients from 19 families worldwide since the description of the disease in 1986 [1–20]. The clinical manifestation varies between mild and severe, involving symptoms such as mental retardation, hyperactivity, behavioural problems, hearing loss, and frequent infections of the skin and respiratory tract. Additional rare clinical signs such as angiokeratomas or severe clinical phenotypes like neonatal epilepsy, hydrocephalus, and spastic paraplegia or Gilles de la Touret syndrome have been reported in some patients. 

So far, only one family with beta-mannosidosis, confirmed by biochemical and enzymatic examination, was described in the Czech Republic in 1990 [5]. The family was of Roma origin with two affected siblings. The girl suffered from severe psychomotor retardation, bone deformities, face dysmorphology - gargoylism, recurrent skin and respiratory infections and died due to bronchopneumonia and sepsis aged 20. The symptoms of her brother were milder but he presented with gargoyle facial dysmorphology, mental retardation, hearing impairment and recurrent infections.

Biochemical diagnostics confirmed the typical disaccharides (primarily Man (beta1 → 4) GlcNac) in the urine of affected patients and a decreased activity of the beta-mannosidase enzyme in different cells, particularly in leukocytes [21, 22]. As usual in metabolic autosomal recessive diseases, the residual activity of the enzyme is very low in affected patients and about half the normal value in heterozygous parents [5].

The human *MANBA* gene encoding the beta-mannosidase enzyme was actually described in the same Czech Roma family in 1998 [19]. The affected siblings had a homozygous c.2158-2A>G variant and their parents were heterozygotes for the same pathogenic variant. Moreover, RNA analysis of the affected siblings showed that the c.2158-2A>G variant is a splicing variant and the activation of the cryptic splice site results in two aberrant products from the patient’s RNA. The first, larger product, lacking 172 bp, results in a frameshift and stop codon that truncates the predicted protein by 155 residues. The second, shorter product, lacks 258 bp and is presumably an exon skipping event [19].

The second family carrying the identical splicing variant c.2158-2A>G was recently reported in a Roma patient from Hungary who presented with hearing loss and mental retardation [23].

Roma known formerly as Gypsies are an ethnic minority group with a high rate of endogamy resulting in a higher occurrence of specific homozygous variants and unique diseases [24–27]. The Roma originate from South Asia and migrated towards Europe between the 5th and tenth century [28, 29]. Therefore, some ancestral founder pathogenic variants were brought from Asia, other private variants arose among the isolated Roma subpopulations in Europe and others were gained by the admixture with other European populations [30, 31].

The two most common variants known to cause hereditary hearing loss among Roma have been identified: the first is in the *GJB2* gene, c.71G>A (p.W24*), which is also found in India, and the other is c.1331+2T>C in *MARVELD2,* described originally in Pakistani families [31, 32].

Both are ancestral founder variants originating in Asia before the Roma migrated to Europe. The average carrier rate of c.71G>A (p.W24*) among the European Roma ranges between 4 and 5% [31]. The average carrier rate of *MARVELD2* is 0.5–2.24% for some European Roma groups [32, 33].

No other frequent cause of deafness has been reported among Roma until now. Here we present a founder variant, causing the rare lysosomal storage disease beta-mannosidosis, presented with hearing loss and mental retardation in Czech and Slovak Roma patients. The detected carrier rate is 3.8% among Czech and Slovak Roma populations. In total, ten Roma patients of Central European origin were found to be homozygous for the *MANBA* c.2158-2A>G pathogenic variant.

## Methods

### Patients recruitment

Altogether **250 hearing-impaired Roma patients with unknown cause** of hearing loss (9 Czech (CZ) and 241 Slovak (SK) patients) were examined. From Slovakia, 187 Roma hearing loss patients with unknown cause of hearing loss were collected from 102 families during the period from 2010 to 2019 and the samples originated from Ear, Nose and Throat departments, schools for hearing – impaired children and from isolated Roma communities. An additional 54 Roma patient samples from Slovakia were collected at schools for hearing-impaired children as part of an earlier research project [34].

All hearing loss patients were previously tested by Sanger sequencing of the *GJB2* (NM_004004.5) exon 2 and 187 of them for the variant c.1331+2T>C (intron 4) in *MARVELD2* (NM_001038603.2); no biallelic variants were detected.

An additional group of **59 hearing loss Slovak Roma patients from 39 families, with already known causal biallelic variants in other deafness genes**, were also tested for the potential presence of the *MANBA* c.2158-2A>G pathogenic variant.

In order to detect the cause of their hearing loss, all subjects, or their legal representatives, gave informed consent for the genetic examination.

### Controls

To examine the carrier rate (frequency of heterozygotes) for the pathogenic *MANBA* variant c.2158-2A>G among hearing Roma population, **345 Roma samples** originated from two cohorts were selected. The first cohort comprised 132 Czech unrelated Roma samples without hearing loss and was selected from families collected with the following diagnoses: congenital myasthenic syndrome; hereditary motor and sensory neuropathy type Lom and Russe; and anonymized. The second cohort of 213 anonymized unrelated Slovak Roma samples without hearing loss came mainly from isolated Roma settlements in different parts of Slovakia.

### DNA analysis

Whole exome sequencing was performed in nine unrelated Czech patients with SureSelect Human all Exon XT v6 kit on Illumina Hiseq4000. The raw fastq data were analysed with SureCall (Agilent,Santa Clara CA, USA) and vcf was uploaded to Ingenuity Variant Analysis (IVA, Qiagen, Redwood City CA, USA) for annotation [35]. First, the variants in the virtual panel of genes reported to be associated with nonsyndromic autosomal recessive or X linked hearing loss (82 genes) were viewed. A second evaluation was focused on variants reported as disease-causing in the HGMD® Professional database (Qiagen,Redwood City CA, USA) or pathogenic/likely pathogenic in ClinVar [36, 37].

In the group of 241 Slovak Roma patients with unknown hearing loss and in the 59 Slovak Roma patients with other known causes of hearing loss, the real time PCR allelic discrimination TaqMan Assay (ThermoFisher Scientific, USA) for c.2158-2A>G variant in *MANBA* gene (NM_005908.3) was examined.

In controls, the variant c.2158-2A>G in *MANBA* gene was tested with custom designed probes for real time PCR allelic discrimination TaqMan Assay (ThermoFisher Scientific, USA).

The presence of the *MANBA* variant c.2158-2A>G detected by WES or real time PCR was confirmed by Sanger sequencing of exon 16 and an adjacent intronic part of the *MANBA* gene. In all detected hearing loss heterozygotes for the *MANBA* variant, all 17 coding exons of the *MANBA* gene were Sanger sequenced in order to exclude a second possible pathogenic variant.

### Clinical evaluations

The clinical investigations were made up from findings sent by the referring physicians. The examination of hearing loss was carried out by various methods (see the results section), with respect to the subjects age and their limited ability to cooperate during the examination.

## Results

Among the 250 Roma patients with an as yet unclarified cause of hereditary hearing loss, nine patients from seven families were found to be homozygous for the pathogenic variant c.2158-2A>G in the *MANBA* gene. Interestingly, one additional homozygote for *MANBA* c.2158-2A>G was also detected in a group of 59 Roma patients with an already clarified cause of hearing loss, namely in a homozygote for *GJB2* c.71G>A (p.W24*) variant. The *MANBA* c.2158-2A>G variant in homozygous patients with hearing loss was also found to cause moderate mental retardation, in line with findings in previously reported beta-mannosidosis patients.

**Fig. 1:**
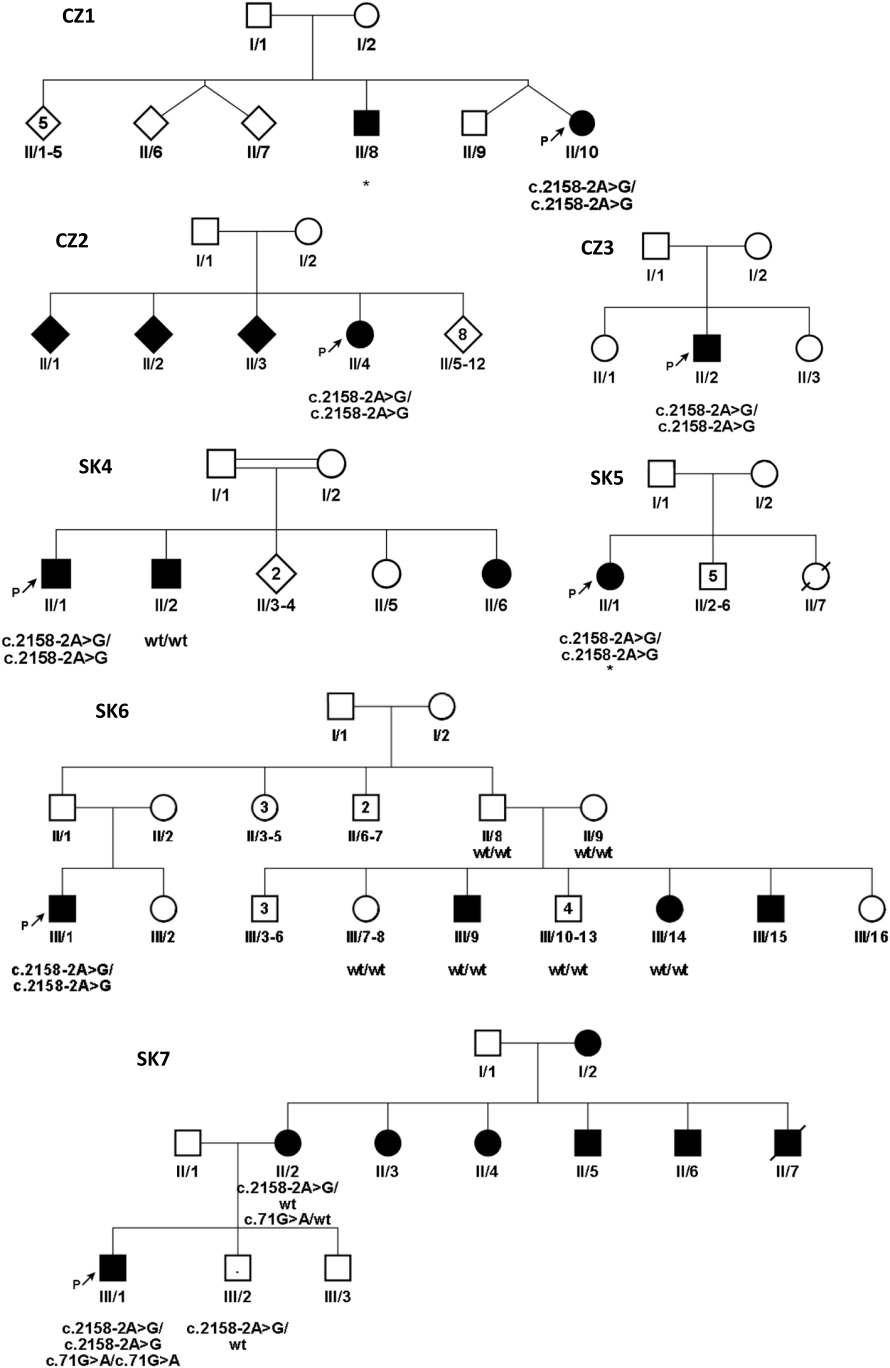
Pedigrees. Pedigrees from Czech (CZ) and Slovak (SK) families – probands and detected variants are marked. The square symbol is for men, circle for women and clinically affected members with hearing loss are filled-in black, rhomb shape – unknown gender, the number in inside the symbols represents the number of siblings. Persons tested genetically for the *MANBA* c.2158-2A>G variant are indicated in pedigrees as c.2158-2A>G or wt – wildtype. * - Patients with beta-mannosidosis diagnosed by biochemical and enzymatic examination. P - indicates the proband, the first affected family member referred for genetic examination. The point in inside the symbol represents the carrier of the pathogenic variant. Given the complexity of family SK7 relationships, we present only the relevant part of the pedigree. The pedigree of family SK8 is missing, we only know that all three are from one family, either siblings or cousins

In the initial three patients from three different Czech families (Fig. 1 - CZ1 – CZ3), the homozygous pathogenic variant c.2158-2A>G was detected by WES. A virtual panel of deafness genes was initally used for WES data filtering, but no pathogenic or likely pathogenic variants were detected (according to ACMG classification) [38]. The second stage filtering from all examined genes led to the identification of the homozygous c.2158-2A>G variant in *MANBA* in all three patients. No other variant either previously reported or classified as pathogenic/likely pathogenic could explain the cause of hearing loss in these patients.

From the group of 241 Roma patients with an unknown cause of hearing loss, six Slovak Roma patients from four families (Fig. 1 - SK4 – SK6, SK8, the pedigree of family SK8 is missing, we only know that all three are from one family, either siblings or cousins.) were among those found to carry the homozygous c.2158-2A>G *MANBA* variant. One additional homozygote for the c.2158-2A>G *MANBA* variant was detected in a group of 59 hearing loss Roma with known variants (Fig. 1 - SK7 – III/1). In the family SK7, three heterozygotes were detected, two in normal hearing siblings (SK7 – III/2 + individual not showed in the pedigree) and one in a hearing-impaired mother (SK7 – II/2), but in this case no second pathogenic *MANBA* variant was found. In the group of 241 hearing-impaired Slovak patients, a total of 13 heterozygotes for c.2158-2A>G were detected. In this patient cohort no second pathogenic variant was detected by Sanger sequencing of all coding exons of the *MANBA* gene.

Five heterozygotes and no homozygotes for the c.2158-2A>G variant were detected in 132 Czech Roma controls without hearing loss, resulting in a 1.89% allele frequency of c.2158-2A>G and a carrier rate (frequency of heterozygotes) of 3.79%. In the group of 213 Slovak Roma controls without hearing loss, eight heterozygotes and no homozygotes were detected with an allele frequency of 1.88% and a carrier rate (frequency of heterozygotes) of 3.76% (Table 1).

**Table 1:** Frequencies of three Roma prevalent hearing loss variants in gnomAD

gnomAD allele frequencies	***MANBA*** c.2158-2A>G	***GJB2*** c.71G>A, p.W24*	***MARVELD2*** c.1331+2T>C
	rs772852668	rs104894396	rs772048719
all	**0.0024%**	**0.052%**	**0.0040%**
South Asian population	**0%**	**0.44%**	**0.020%**
genotypes - het: wt	0: 15303	134: 15174	6: 15302
European Non-Finish population	**0.0018%**	**0.0063%**	**0.0026%**
genotypes - hom:het:wt	0: 2: 56692	1: 6: 63872	0: 3: 56781
Latino population	**0.012%**	**0.0028%**	**0%**
**Allele frequencies detected in this report**			
CZ genotypes - het: wt	**1.89%** 5: 127 (3.79%)	**Not tested**	**Not tested**
SK genotypes - het: wt	**1.88%** 8: 205 (3.76%)	**Not tested**	**Not tested**

Legend – gnomAD [39] allele frequencies and genotypes for prevalent Roma variants in *MANBA*, *GJB2* and *MARVELD2* are shown. All populations, in which the frequency was detected, are shown except for the c.71G>A (p.W24*) where one more frequency is listed (OTH - other populations, 4 heterozygotes were detected). The frequency and numbers of individual genotypes are listed for the complex overview

*CZ -* Czech Roma population, *SK* - Slovak Roma population, *Het* - heterozygous for pathogenic allele, *hom -* homozygous for pathogenic allele, *wt -* wildtype, both alleles are reference alleles, frequencies are in bold

### Clinical findings

In family CZ1, two siblings were affected by beta-mannosidosis. WES examination revealed the pathogenic variant in homozygous state in the affected girl (CZ1 - II/10) and independent metabolic disease screening followed by biochemical examination confirmed the disease in her older brother (CZ1 - II/8) long before a genetic examination. In both siblings delayed motor milestones were reported. Both started to walk independently at two years and say their first words at four and a half. (Table 2 – clinical findings). The girl has hypertelorism, broad nose root, strabismus and a smaller stature (Table 2). The boy (CZ1 - II/8) suffered from recurrent otitis media and preschool age bronchitis which improved after adenoidectomy. Both siblings have a medium degree of mental retardation as well as behavioural disorders and prelingual bilateral hearing loss. Brainstem Auditory Evoked Potentials were bilaterally abnormal and severe hearing loss was detected in the girl (CZ1 - II/10). A biochemical examination of the boy revealed oligosaccharides in his urine and a decreased beta-mannosidase activity in leucocytes (1.65 nmol/h/mg versus control 185 nmol/h/mg). DNA from the boy and the parents was not available for examination, but from the clinical findings we may assume that he is also homozygote for the same pathogenic allele as his sister, and that both parents are heterozygotes. Because we cannot examine the c.2158-2A>G *MANBA* variant, this patient was excluded from the results of the genetic examination, but his clinical features are included in the Table 2 and in the discussion.

**Table 2:** Clinical findings in patients with beta-mannosidosis due to *MANBA* (c.2158-2A>G) homozygous variant

Family - patient	Reported by	Hearing loss	Mental retardation	Infectious manifestations	Facial dysmorphism	Skeletal defects	Other symptoms	Variant
**CZ1 – II/8**	this report	prelingual hearing impairment	moderate mental retardation – behavioral disorder - aggressiveness	frequent otitis and bronchitis in preschool age	no	no	no	NO DNA testing – biochemical and enzymatic detection of beta-mannosidosis
**CZ1 – II/10**	this report	severe prelingual hearing impairment	moderate mental retardation – behavioral disorder	no	hypertelorism, broad nose root	no	strabismus, short stature 149 cm at the age of 12 y	*MANBA* (c.2158-2A>G) hom
**CZ2 – II/4**	this report	moderate prelingual hearing impairment	moderate mental retardation	no data	no data	no data	no data	*MANBA* (c.2158-2A>G) hom
**CZ3 – II/2**	this report	moderate hearing impairment	mental retardation	frequent bronchitis in preschool age	no data	no data	no data	*MANBA* (c.2158-2A>G) hom
**SK4 – II/1**	this report	severe to profound prelingual hearing loss – PTA 90 dB for R, 70 dB for L	moderate mental retardation with autistic features	recurrent bronchopneumonia, gastroenteritis, otitis media, conjunctivitis	no data	growth retardation	urinary incontinence, strabismus, anemia, hyperphagia with normal body weight	*MANBA* (c.2158-2A>G) hom
**SK5 – II/1**	this report	moderately severe prelingual progressive hearing loss	moderate mental retardation with increased impulsivity and psychotic manifestations	no	broad nose	no data	short stature (153 cm at the age of 26y), hirsutism	*MANBA* (c.2158-2A>G) hom
**SK6 – III/1**	this report	moderate prelingual hearing loss – PTA 45 dB	no data	no data	no data	no data	no data	*MANBA* (c.2158-2A>G) hom
**SK7 – III/1**	this report	congenital hearing loss	mental retardation with autistic features	no data	no data	no data	convergent strabismus	*MANBA* (c.2158-2A>G) hom
**SK8–1**	this report	hearing impairment	no data	no data	no data	no data	no data	*MANBA* (c.2158-2A>G) hom
**SK8–2**	this report	hearing impairment	no data	no data	no data	no data	no data	*MANBA* (c.2158-2A>G) hom
**SK8–3**	this report	hearing impairment	no data	no data	no data	no data	no data	*MANBA* (c.2158-2A>G) hom
**Fam 1 - II/8**	[5]	not mentioned	severe mental retardation – auto mutilations – tearing of hairs leading to focal alopecia	recurrent skin and respiratory infections	gargoyl facies – hypertelorism, macroglossia, gingival hyperplasia, short neck	deformities of the thorax, lumbar hyperlordosis and nanism	severe anemia	*MANBA* (c.2158-2A>G) hom
**Fam 1 - II/2**	[5]	hearing impairment	moderate mental retardation, occasional aggressive behavior.	recurring erysipelas-like skin changes and respiratory infections	slight hypertelorism and thick lips	no	no	*MANBA* (c.2158-2A>G) hom
**Fam 6005 – II:1**	[23]	moderate to profound hearing impairment	intellectual disability, behavioral problems – attention-deficit/hyperactivity disorder	respiratory inflammations treated as cystic fibrosis	not mentioned	not mentioned	no	*MANBA* (c.2158-2A>G) hom

In family CZ2, four siblings are affected by hearing loss, but only one (CZ2 - II/4) was available for genetic testing (Fig. 1). Prelingual hearing loss, with behavioural disorders and moderate mental retardation dominated the clinical picture. According to audiometric testing the level of hearing impairment was moderate (audiogram 500 Hz – 20 dB, 2000 Hz – 50 dB, 4000 Hz – 60 dB), the speech reception threshold (SRT) was 75 dB, and 100% word discrimination was at 90 dB.

Hearing loss in patient (II/2) from family CZ3 was detected at three years of age and is classified as moderate (58.8% according to Fowlers scale). First neurological examination at the age of 7 revealed mental retardation and patient is in dispensary care for mentally-retarded patients.

In family SK4 with confirmed consanguinity between the parents, at least three out of five siblings suffer from early onset sensorineural hearing loss. Their mother is mentally retarded, as is the patient (SK4 – II/1) and his younger sister though to a moderate degree. Only two subjects were available for DNA analysis. The patient (SK4 – II/1) is a homozygous boy with reported developmental and growth delay, but with a normal birth size (3100 g, 49 cm); recurrent respiratory and gastrointestinal infections, urinary incontinency of 3rd degree until at least six years of age, strabismus, abnormal appetite without obesity, severe to profound hearing loss, and moderate degree of mental retardation with autistic behaviour. His brother (SK4 – II/2) has moderately-severe hearing loss and thought to have mild intellectual disability without any other reported disease symptoms. The c.2158-2A>G variant was not detected in this subject. Based on available data, children from this family are dispersed among several orphanages due to the poor social-economic conditions of the parents.

**Fig. 2:**
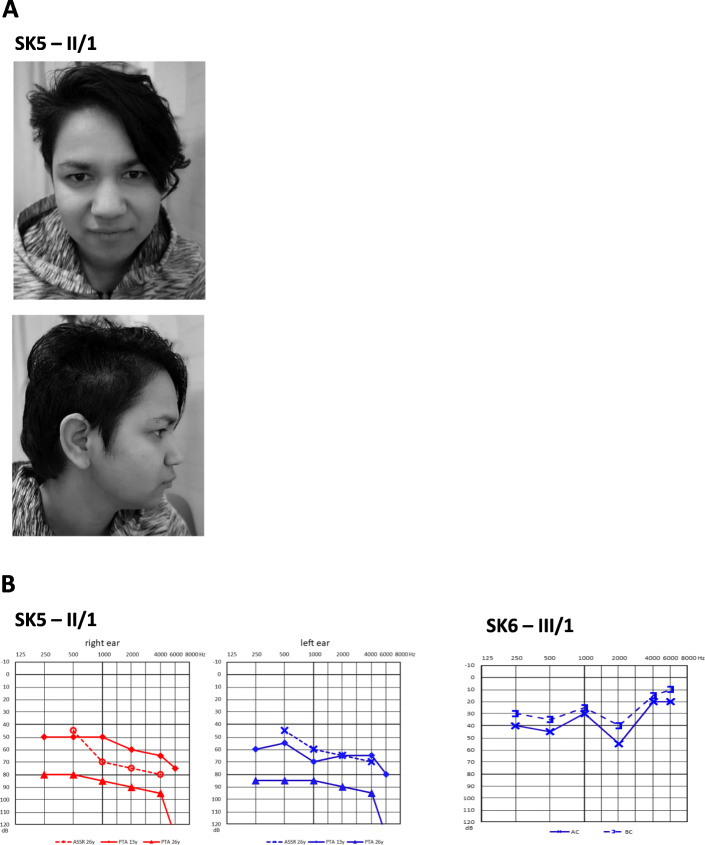
Photos and audiograms. **a**– Photos of affected patient SK5 – II/1 the broad nose is visible, no angiokeratomas on the patient were visible – not shown. **b** – Audiograms from families SK5 and SK6. The audiogram in SK5 - II/1 – dashed line represents ASSR (objective estimation of the hearing threshold), full lines are pure tone, audiometric curves at the age of 13 and 26 years. Note the discrepancy between pure tone audiometry and ASSR thresholds measured at the same age (26 years) which might result from mental retardation. In patient SK6 - III/1 the audiogram for only the left ear is presented; it was not possible to examine reliably the right ear due to noncompliance of the subject with mental retardation

The 26-year-old female patient from family SK5 (II/1) has prelingual hearing loss with gradual progression based on serial audiograms and flat to gently sloping audiometric curves (Fig. 2a and b). She also suffers from moderate mental retardation with behavioural changes (impulsivity) and psychotic symptoms. She has short stature (153 cm) and the only apparent facial feature is a broad nose. The subject is otherwise healthy with a negative clinical history of recurrent infections. The beta mannosidase activity in peripheral blood leucocytes was undetectable (0.00 nmol/h/mg, reference values 65–180 nmol/h/mg) which suggests deficit without any residual activity. The biochemical analysis confirmed typical saccharide profile in urine, i.e. free oligosaccharides were identified. The highest peak values corresponded to Man-GlcNac (518 Da) and the presence of trisaccharides NeuAc-Man-GlcNac (879 Da) and pentasaccharide (1328,6 Da) do confirm the accumulation of free oligosaccharides in urine resulting from inactive beta-mannosidase. This patient comes from an incomplete family and lives in an institution for the mentally disabled. According to the available clinical files, there are no other affected family members.

The homozygous patient from family SK6 (III/1) has prelingual hearing loss of moderate degree based on absent otoacoustic emissions and audiometric evaluation (Fig. 2 b). However, reliable pure tone thresholds were only recorded for the left ear due to the subject’s poor collaboration. The subject originates from an isolated Roma community in eastern Slovakia and was recruited during a field study that focused on hereditary hearing loss, thus we do not have any precise data on his intellectual status. All other first-degree relatives refused genetic testing.

In family SK7, recruited in an isolated Roma community in eastern Slovakia (different from the previous case), we identified a mother and child affected by hearing loss (Fig. 1 – SK7-II/2, III/1). The boy (SK7 – III/1) carrying the biallelic c.2158-2A>G variant was reported to have congenital deafness and mental retardation with autistic features. Regarding other pathologies, he also had apparent convergent strabismus. At the age of seven, he was not able to understand instructions required to undergo an audiometric assessment. Interestingly, this subject was detected to have two different autosomal recessive causes of hearing loss since besides having a homozygous c.2158-2A>G *MANBA* variant he is also a homozygote for the c.71G>A (p.W24*) variant in *GJB2*. His hearing-impaired mother (SK7 – II/2) is a heterozygote for the c.2158-2A>G variant and she had several other relatives suffering from hearing loss, who were not available for genetic testing. This family case confirms and highlights the high frequency of both pathogenic variants in the Roma population.

## Discussion

The first, and to date, only family with beta-mannosidosis in the Czech Republic was reported in 1990 by Kleijer et al. [5]. Despite the extremely rare worldwide occurrence of beta-mannosidosis, we were able to detect 11 new patients from eight families from the Czech Republic and Slovakia with this disease, all with hearing loss and all of Roma ethnicity; 10 of them were homozygous for the same variant c.2158-2A>G in *MANBA* and one was diagnosed with biochemical and enzymatic detection of beta-mannosidosis.

Storage disorders are rare and very heterogeneous diseases. The prevalence of beta-mannosidosis is 0.16 per 100,000 [40]. All 23 patients reported to date carry different pathogenic variants in the *MANBA* gene and, except for the siblings, no patient shares the same variant with the other. There is only one exception; the c.2158-2A>G *MANBA* variant (the Human Gene Mutation Database [37], which was reported in one Czech Roma family and in one other Roma patient from Hungary reported in 2019 [19, 23]. We describe ten new patients from eight Roma families from the Czech and Slovak Republics with the c.2158-2A>G variant in the homozygous state. We also detected a carrier frequency of 3.77% in the Czech and Slovak normal hearing Roma population. However, we have not detected this variant among 289 Czech and Slovak non-Roma patients with different diagnoses collected for WES (in-house databases).

Due to various reasons, cultural, behavioural and social habits, the Roma population is very endogamous and may explain the various prevalent and ethnic-specific variants responsible for several different diseases that exist in this population. Some genes are known to be only affected in the Roma ethnicity. Variants c.442C>T (p.Arg148*) in the *NDRG1* gene (NM_006096.3) or c.-249-3818G>C in the *HK1* gene (NM_033498.2) are an important causes of hereditary neuropathy. Similarly, the variant c.863 + 389C>T in the *CTDP1* gene (NM_004715.4) is responsible for Congenital cataract facial dysmorphism and demyelinating neuropathy (CCFDN, OMIM 604468), but all these diseases are found only in the Roma population [24, 25, 27]. Other variants in these genes are not responsible for the neuropathy in any other ethnic group. Even more common is the case when variants in a certain gene are associated with a disease and different variants occur or are prevalent in several different populations. Therefore we may observe ethnic-specific variants like the c.71G>A (p.W24*) in the *GJB2* gene, which is responsible for a substantial number of hearing loss cases in the Roma population, and in Indian cases as well [31]. On the other hand, the variant c.35delG in the same gene predominantly causes hearing loss in the non-Roma Caucasian population, but can also be detected in the Roma population [31]. We may suppose that *MANBA* gene belongs to this group, as the c.2158-2A>G variant also shows ethnic specificity and is prevalent in the Roma population.

If we focus on the most prevalent variants causing hearing loss in the Roma population, there are only two other variants known so far, the c.71G>A (p.W24*) in *GJB2* and the c.1331+2T>C in *MARVELD2*. The detected carrier frequency 3.8% for c.2158-2A>G in *MANBA* is very similar to the average carrier rate of c.71G>A (p.W24*) in *GJB2* among the European Roma, which is in the 4–5% range [31]. Based on our results, the *MANBA* variant seems to be a more important and more frequent cause of hearing loss compared to *MARVELD2*. The c.1331+2T>C in *MARVELD2* has an average carrier rate of 1% for Czech Roma compared to 2.24% for Slovak Roma and 0.5% for Hungarian Roma [32, 33].

If we try to trace the origin of the *MANBA* variant, we have to consider the following facts: our findings show the variant c.2158-2A>G to be an important cause of hearing loss in the Czech and Slovak Roma, but not in non-Roma European populations. This suggests that the variant did not arise from an admixture with the majority European population such as with c.35delG in *GJB2*, but either originated in India before the Roma left, or arose among the isolated Roma minority on the European continent. According to Schrauwen et al. the multidimensional scaling analysis (MDS) and admixture analysis showed a more pronounced South Asian background for two individuals carrying the *GJB2* (c.71G>A, p.W24*) and *MANBA* (c.2158-2A>G) variants, based on the similarity with individuals from the Punjab province (SAS – South Asian). Unfortunately, no patient with simultaneous *MARVELD2* variant was detected in the paper and therefore it could not be added to the admixture and MDS analysis [23]. However, the comparison of frequencies from gnomAD [39] gave different results (Table 1). No allele for c.2158-2A>G (*MANBA*) was detected in the SAS population, compared to 134 heterozygotes for c.71G>A (p.W24*) (*GJB2*) and six heterozygotes for c.1331+2T>C in *MARVELD2*. The almost opposite is true for the NFE (European, Non-Finish) population, where two heterozygotes for c.2158-2A>G in *MANBA*, six heterozygotes and one homozygote for c.71G>A (p.W24*) (*GJB2*) and three heterozygotes for c.1331+2T>C in *MARVELD2* were detected (Table 1). The carrier frequencies of the prevalent variants in SAS: NFE populations are: 0:0.0018% for *MANBA*, 0.875%:0.009% for *GJB2* and 0.039%:0.005% for *MARVELD2*. In the both prevalent variants from Asia (*GJB2* and *MARVELD2*), the frequency of SAS is by 1–2 orders higher compared to NFE. Therefore, we assume that the prevalent *MANBA* variant arose among the Roma population only after they left India. Only the frequency investigation of this variant in individual Roma subpopulations over Europe can answer the commonality of this variant in other European countries. Moreover, the detected carrier frequency of *MANBA* in the Czech and Slovak Roma population is 925x higher compared to the carrier frequency in the NFE population gnomAD [39] (Table 1).

So far, including this paper, 14 patients of Roma origin manifesting beta-mannosidosis have been described (Table 2). All 14 patients were homozygous for the sequence variant c.2158-2A>G in the *MANBA* gene. There is probably and even greater incidence of beta-mannosidosis in the patients (8 families) included in this study, however, samples were not available for all family members with hearing loss. The most prominent and common clinical manifestations are mental retardation reported in ten patients and hearing loss mentioned in 13 patients (Table 2). The mental retardation is rather moderate, but frequently additional features like behavioural problems, aggressiveness and autistic features are associated. The degree of hearing loss is moderate and moderately severe. Other rare symptoms presented in less than half of reported patients are respiratory and skin infections reported in five patients, short stature reported in four and hypertelorism reported in three [5, 23].

The hearing in patient SK5 - II/1 displays different levels of hearing loss when the results of subjective (pure tone audiometry) and objective (ASSR) methods are compared. The higher values of PTA thresholds compared to ASSR could be at least partially explained by limited cooperation due to mental retardation. Therefore, both subjective and objective methods for audiometric examination should be used in *MANBA* related patients to properly evaluate the degree of hearing impairment.

Interestingly, in several patients with hearing loss from SK families (Fig. 1 – SK4, SK6, SK7), the variant c.2158-2A>G was not detected. In addition, in the SK7 family, the mother (II/2) is affected with hearing loss, but the genetic cause of her disease is still unknown, nevertheless she is a carrier of two pathogenic variants, both of which caused recessive hearing loss in her child (c. 2158-2A>G in *MANBA*, c.71G>A in *GJB2*). A condition for recruitment into this study was the presence of hearing loss in Roma patients; hearing loss is a heterogeneous disease, where over 100 genes have been reported [41]. We have shown that genetic heterogeneity is present even in the endogamy groups of the Roma from Slovakia, where causes other than c.2158-2A>G must be present in order to explain hearing loss in some individuals.

The clinical manifestation of beta-mannosidosis is variable and genotype/phenotype correlation cannot be completely traced. Bedilu et al. tried to identify clinical signs in null variants causing beta-mannosidosis in two families [11]. In conclusion, they found that only the severity of the variant and the associated near-zero enzyme activity did not explain the variability of clinical manifestations in null variants, and that another component must be involved [11]. Although it was considered that the combination of null and missense variants would explain the milder phenotype of the disease associated with residual activity of the enzyme itself, it was not proven. The combination of a null variant with a missense variant makes the enzyme activity almost null and an explanation for the milder phenotype could be due to epigenetic or environmental factors [15]. The phenotypes of Roma patients with the c.2158-2A>G variant comprise mainly moderate to severe hearing loss and moderate mental retardation. Previous analysis of the splicing variant c.2158-2A>G revealed two aberrant shorter products from the patient’s RNA, therefore it is not a null variant; and, given some gene products are present, it could be the result of the milder phenotype of beta-mannosidosis expressed in Roma patients. However, a study of the pathophysiology of this specific variant is beyond the scope of this paper.

## Conclusion

The detected c.2158-2A>G variant in *MANBA* is another important ethnic-specific variant threatening the Roma population in Central Europe. This variant, in a homozygous state, causes the disease beta-mannosidosis and is an important and previously unknown cause of hereditary moderate to severe hearing loss in Roma patients associated with mental retardation. Based on the high frequency of carriers, we expect a higher frequency of those affected by beta-mannosidosis among the Roma. Due to the main feature of the disease, i.e. mental retardation, most of the affected subjects may suffer from an even higher degree of social isolation than the rest of the Roma minority and, with poor access to adequate healthcare, go undetected. The second important clinical feature that could be overlooked is hearing loss, especially when it is only moderate; it is a result of either poor patient compliance or a failure to associate hearing loss with this phenotype. Beta-mannosidosis is an extremely rare disease that can be detected easily, biochemically and genetically, yet there is no treatment. Due to the high frequency of disease carriers in the Roma population, we suggest offering carrier screening to interested Roma individuals. Ultimately, we recommend routine genetic testing, at least in Central Europe, of this ethnically-specific pathogenic variant in all Roma with unexplained hearing loss and mental retardation.

## Supplementary information

**Additional file 1.** Supplementary file – table 1_ genes included in the virtual gene panel for early/prelingual hearing loss.

## Data Availability

The datasets supporting the conclusions of this article are included within the article.

## Abbreviations

WES: Whole exome sequencing
gnomAD: The Genome Aggregation Database
SAS: The South Asian population
NFE: The Non-Finnish European population
HGMD: The Human Gene Mutation Database
