# Supplementary material for: Variant c.2158-2A>G in MANBA is an important and frequent cause of hereditary hearing loss and beta-mannosidosis among the Czech and Slovak Roma population- evidence for a new ethnic-specific variant
Source: Orphanet J Rare Dis. 2020 Aug 26;15:222. doi: 10.1186/s13023-020-01508-3 (PMC7448337; doi:10.1186/s13023-020-01508-3)
Supplement: Supplementary file 1 — Additional file 1. Supplementary file – table 1_ genes included in the virtual gene panel for early/prelingual hearing loss. [file 13023_2020_1508_MOESM1_ESM.docx]

Supplementary file – table 1_ genes included in the virtual gene panel for early/prelingual hearing loss.

| DFNB1A | GJB2 |
| --- | --- |
| DFNB1B | GJB6 |
| DFNB2 | MYO7A |
| DFNB3 | MYO15A |
| DFNB4 | SLC26A4 |
| DFNB6 | TMIE |
| DFNB7/11 | TMC1 |
| DFNB8/10 | TMPRSS3 |
| DFNB9 | OTOF |
| DFNB12 | CDH23 |
| DFNB15/72/95 | GIPC3 |
| DFNB16 | STRC |
| DFNB18 | USH1C |
| DFNB18B | OTOG |
| DFNB21 | TECTA |
| DFNB22 | OTOA |
| DFNB23 | PCDH15 |
| DFNB24 | RDX |
| DFNB25 | GRXCR1 |
| DFNB26 | GAB1 |
| DFNB28 | TRIOBP |
| DFNB29 | CLDN14 |
| DFNB30 | MYO3A |
| DFNB31 | DFNB31 |
| DFNB32/105 | CDC14A |
| DFNB35 | ESRRB |
| DFNB36 | ESPN |
| DFNB37 | MYO6 |
| DFNB39 | HGF |
| DFNB42 | ILDR1 |
| DFNB44 | ADCY1 |
| DFNB48 | CIB2 |
| DFNB49 | MARVELD2 |
| DFNB49 | BDP1 |
| DFNB53 | COL11A2 |
| DFNB57 | PDZD7 |
| DFNB59 | DFNB59 |
| DFNB60 | SLC22A4 |
| DFNB61 | SLC26A5 |
| DFNB63 | LRTOMT |
| DFNB66 | DCDC2 |
| DFNB66/67 | LHFPL5 |
| DFNB68 | S1PR2 |
| DFNB70 | PNPT1 |
| DFNB73 | BSND |
| DFNB74 | MSRB3 |
| DFNB76 | SYNE4 |
| DFNB77 | LOXHD1 |
| DFNB79 | TPRN |
| DFNB82 | GPSM2 |
| DFNB84 | PTPRQ |
| DFNB84 | OTOGL |
| DFNB86 | TBC1D24 |
| DFNB88 | ELMOD3 |
| DFNB89 | KARS |
| DFNB91 | SERPINB6 |
| DFNB93 | CABP2 |
| DFNB94 | NARS2 |
| DFNB97 | MET |
| DFNB98 | TSPEAR |
| DFNB99 | TMEM132E |
| DFNB100 | PPIP5K2 |
| DFNB101 | GRXCR2 |
| DFNB102 | EPS8 |
| DFNB103 | CLIC5 |
| DFNB104 | FAM65B |
| DFNB106 | EPS8L2 |
| DFNB108 | ROR1 |
|  | WBP2 |
|  | ESRP1 |
|  | MPZL2 |
|  | CEACAM16 |
|  | GRAP |
|  | SPNS2 |
|  | CLDN9 |
| DFNX1* | PRPS1 |
| DFNX2 | POU3F4 |
| DFNX4 | SMPX |
| DFNX5 | AIFM1 |
| DFNX6 | COL4A6 |
|  | CLPP |
| DFNMYP | SLITRK6 |
